# Mixed gain detector configurations for time-resolved X-ray solution scattering

**DOI:** 10.1107/S1600577524012219

**Published:** 2025-02-06

**Authors:** Morten Lunn Haubro, Joseph Pon, Philip Adam Hart, Kristoffer Haldrup, Tim Brandt van Driel

**Affiliations:** a Department of Physics Technical University of Denmark 2800Kongens Lyngby Denmark; b University of California at Irvine, Irvine, CA92697, USA; c Linac Coherent Light Source SLAC National Accelerator Laboratory 2575 Sand Hill Road Menlo Park CA94025 USA; RIKEN SPring-8 Center, Japan

**Keywords:** XFEL, detector, gain switching, detector non-linearity, diffuse scattering

## Abstract

X-ray detection at X-ray free-electron lasers is challenging in part due to the XFEL’s extremely short and intense X-ray pulses. Experimental measurements are further complicated by the large fluctuations inherent to the self-amplified spontaneous emission process producing the X-rays. At the Linac Coherent Light Source the ePix10ka2M detector offers multiple gain modes, and auto-ranging between these, to increase the dynamic range while retaining low noise. For diffuse scattering techniques, such as time-resolved X-ray solution scattering, where the shape of the scattering pattern largely does not change between exposures, a fixed mix of different gain modes offers many of the same advantages as auto-ranging. We find that configuring individual ASICs in separate gain modes does not impact the intensity linearity of the gain response and has a limited effect on the effective dynamic range in regions with different gain mode settings while avoiding the complexities of auto-ranging. Small (<5%) non-linear gain contributions arise when pixels on the same ASIC are configured in different gain modes. We present a configuration scheme that is designed to select the optimal mixed gain configuration to minimize effects of saturation in the high-/medium-gain region, while maximizing the number of pixels with higher gain to improve the signal-to-noise ratio.

## Abstract 2

The linearity of the intensity response of the ePix10k detector in mixed gain configurations is evaluated for solution phase scattering experiments at the LCLS.

## 1. Introduction

**Figure 1:**
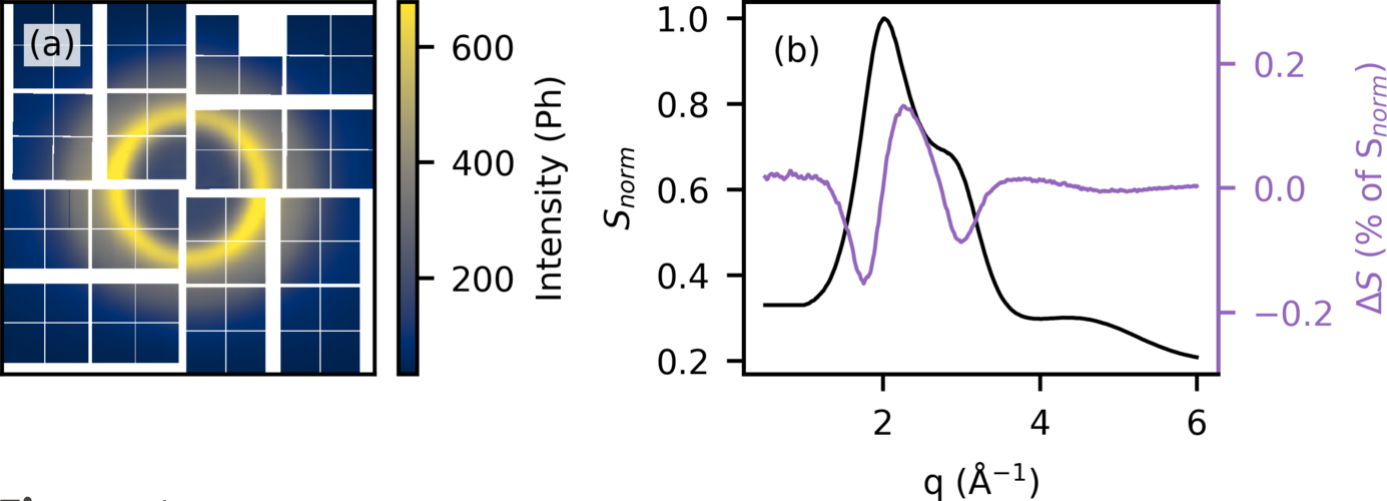
Left: XSS scattering pattern from water with 9.5 keV X-ray photons, measured using the ePix10k detector at the XCS endstation at LCLS. Right: azimuthally averaged normalized scattering pattern, *S*_norm_ (black), and difference scattering, Δ*S*, as a percentage of the total measured intensity (purple). The scattering vector *q* is related to the scattering angle 2θ as *q* = 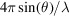, where λ is the wavelength of the X-rays.

Time-resolved X-ray solution scattering (TR-XSS) utilizing a pump–probe approach and X-ray free-electron laser (XFEL) radiation has emerged as a powerful technique for investigating structural dynamics in ultra-fast (10^−15^ to 10^−9^ s) photochemical processes (Gaffney, 2021 ▸; Choi *et al.*, 2022 ▸). Due to its direct structural sensitivity (Jeong *et al.*, 2022 ▸), TR-XSS provides distinctly different information than both optical and X-ray spectroscopy methods. Fig. 1 ▸(*a*) shows an example of an XSS scattering pattern measured on a water sample. Such signals are typically treated by azimuthally integrating the obtained scattering patterns, and difference scattering signals are constructed, isolating the changes to the scattering signal induced by the pump laser. Fig. 1 ▸(*b*) shows a typical difference scattering pattern, arising from solvent heating, normalized to the total signal intensity. Difference scattering signals are typically <1% of the total scattering signal, making TR-XSS extremely sensitive to systematic errors caused by, for example, liquid jet instabilities, self-amplified spontaneous emission (SASE) fluctuations, detector artefacts, or drifts affecting either the X-ray or laser beam. Several different methods have been presented for removing systematic noise, such as parameter-based filtering (*e.g.*X-ray intensity, energy or arrival time), as well as methods for direct subtraction of artefacts, based on singular value decomposition of the detector response (van Driel *et al.*, 2015*a* ▸) or of the difference scattering data (Haldrup, 2014 ▸; Canton *et al.*, 2015 ▸). Accurate detection is furthermore complicated by the large variations in signal intensity, both between exposures, as XFEL pulses can fluctuate drastically in intensity, as well as within each scattering pattern where the per pixel intensity can range from a single to hundreds of photons as seen in Fig. 1 ▸. To accommodate such intensity variations, modern X-ray detectors accommodate several different signal amplification or *gain* modes. Choosing the optimal gain configuration for the detector for a given measurement is, however, challenging as higher gain leads to saturation in the liquid ring and lower gain leads to readout noise limiting the data quality, especially in lower intensity regions of the scattering pattern.

To increase the dynamic range while minimizing the noise, several detectors have been developed with multiple gain settings (Hart *et al.*, 2012 ▸; Redford *et al.*, 2016 ▸). Such detectors often have configurations for fixed single gain modes (detector or modules are configured in the same gain), fixed mixed gain modes (different modules or pixels are configured in different gains), simultaneous gain modes (where multiple gains are read out and the ideal gain is selected during analysis) as well as auto-ranging/auto-switching gains (where pixels switch to a lower gain at a given threshold on a pixel-to-pixel level for each individual frame). While mixed and auto-ranging modes minimize readout noise, there is a risk that they may also introduce detector artefacts in the form of effective pedestals and local non-linearities in the detector response at the gain boundary. These effects can cause deviations of >10% relative to their fixed gain counterparts. In principle, such non-linearities can be removed using the methods described in this manuscript. However, as will be demonstrated in Section 5.2, neighbouring pixels are not entirely independent and so the response of a single pixel not only depends on the intensity on that pixel but also on what gain mode neighbouring pixels are in. In practice, this means that the performance in the *gain switching region* is substantially worse and that, even after corrections, the performance is affected. Use of mixed gain and auto-ranging therefore need to be carefully characterized and considered on an experiment to experiment basis.

Due to the small difference signal magnitude [see Fig. 1 ▸(*b*)], TR-XSS requires a high degree of linearity from the detector response, otherwise systematic errors of the same magnitude as the difference scattering signal are introduced. At the Linac Coherent Light Source (LCLS), the CSPAD detector (Blaj *et al.*, 2015 ▸) was shown to have ∼5% non-linear contributions to the detector response as a result of crosstalk (van Driel *et al.*, 2015*b* ▸). van Driel *et al.* showed that these effects, while problematic, can be removed effectively while retaining both the spatial and intensity distribution of the signal, using the method outlined in Section 3.1. The newer ePix10ka2M detector (Blaj *et al.*, 2019 ▸), here denoted as ePix10k, showed further improvement in detector linearity (van Driel *et al.*, 2020 ▸) which, in addition to improvements in beam stability, made such corrections less vital. The push for higher X-ray energies, better time resolution, and more relevant, weaker scattering sample systems increases the need for pushing the signal-to-noise limit by, for example, utilizing optimized experiment specific gain configurations, such as the ones presented in this work. X-ray detectors are very well characterized and calibrated under generic conditions (flat field and weak illumination), but the specific experimental conditions at XFELs often differ significantly. As the repetition rate of XFELs increases, and it becomes infeasible to store the raw detector images, understanding detector response under the relevant experimental conditions becomes even more critical. Weaker difference scattering signals, higher X-ray repetition rate, as well as the use of novel multi-gain detector configurations makes characterization and correction of the gain response under experimental conditions critical.

## 2. The ePix10k2M detector

**Figure 2:**
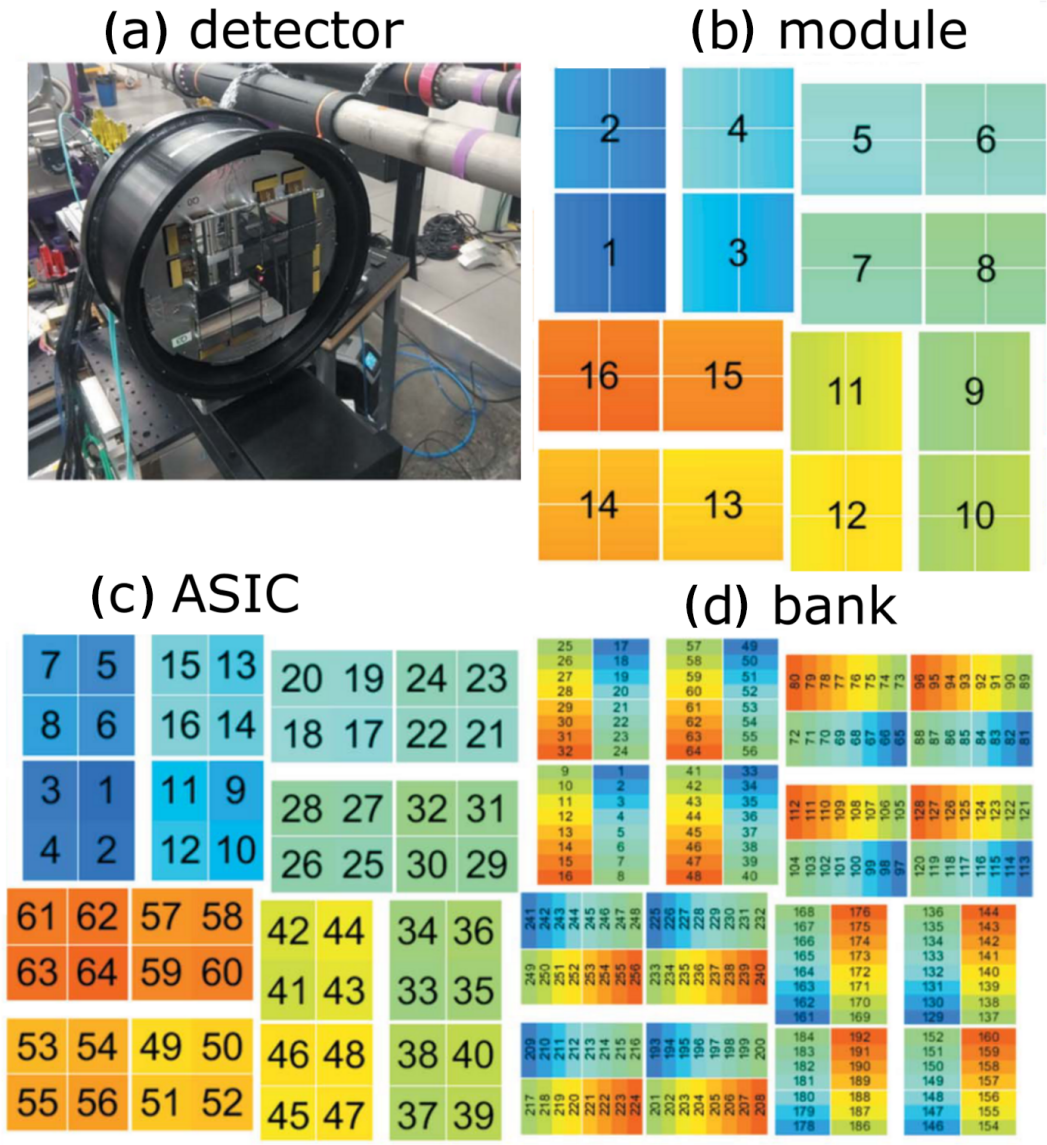
Layout of the ePix10k detector, showing the modular assembly comprising the detector of modules, ASICs and banks (van Driel *et al.*, 2020 ▸).

The ePix10k detector (van Driel *et al.*, 2020 ▸) at the XCS (X-ray Correlation Spectroscopy) endstation (Alonso-Mori *et al.*, 2015 ▸) is a hard X-ray integrating 2D pixel array detector, optimized for high dynamic range at ∼10 keV. Fig. 2 ▸ shows the ePix10k detector (*a*), and the subdivisions of the detector surface (*b*–*d*). The detector consists of four quadrants; each quadrant is subdivided into four modules (*b*), and each module consists of four application specific integrated circuits (ASICs), bump-bonded to one silicon sensor (*c*), each ASIC containing four detector banks (*d*). The silicon sensors have a thickness of 500 µm. Each module is mounted on a printed circuit board that supplies voltages and each quadrant is supplied and read out independently.

**Figure 3:**
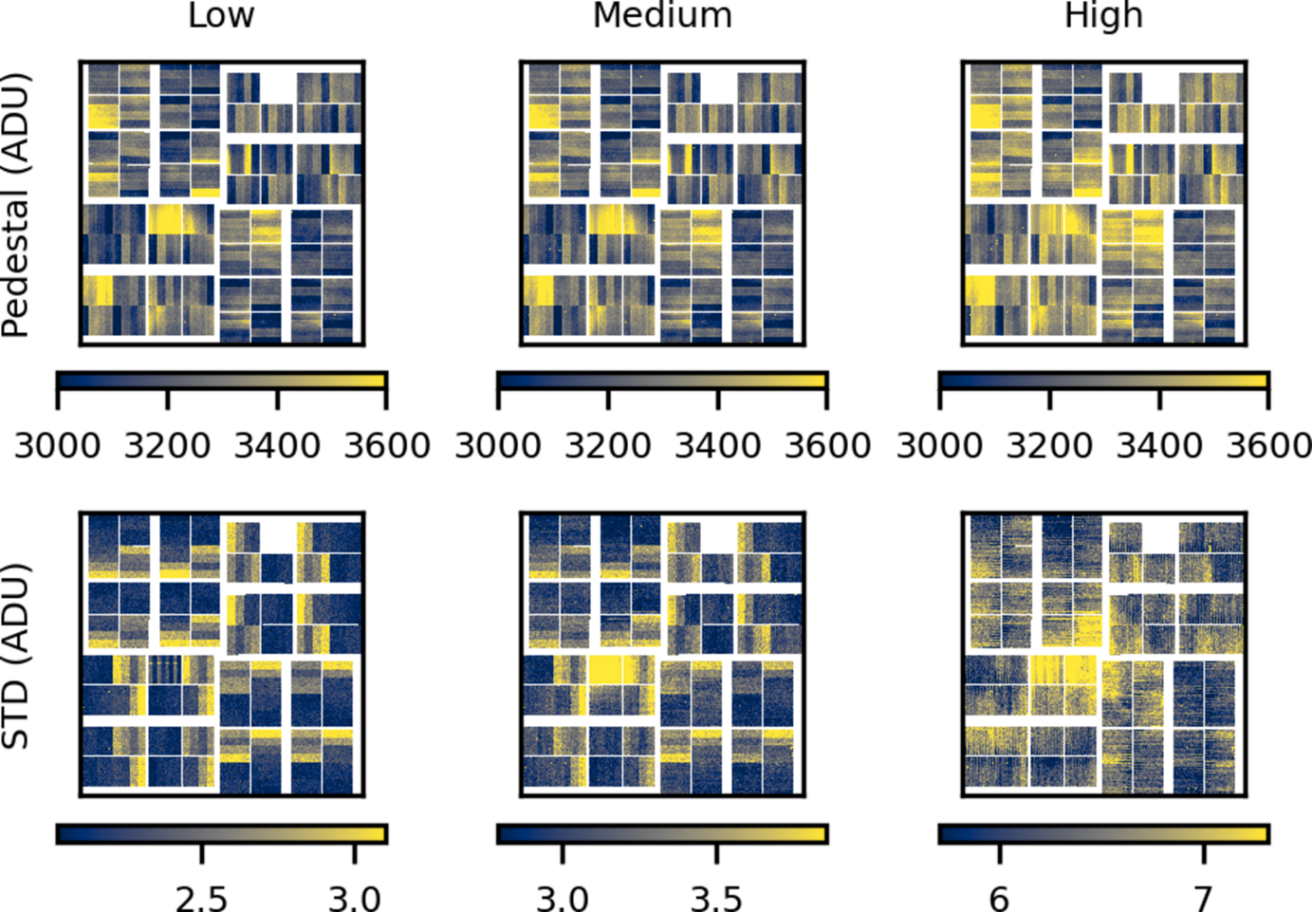
Pedestal in all three gain modes (top row) and the corresponding standard deviation or readout noise (bottom row).

To accommodate the large dynamic range requirements of XFEL experiments, the detector has three fixed gain modes (high, medium, low) as well as two auto-ranging gain modes (high-to-low, medium-to-low) that can be configured on a pixel-to-pixel or an ASIC level. The detector dark level, the pedestal, is measured as the mean of a set of unexposed detector images. The pedestal is measured several times per 12 h shift to account for any temperature drifts or short-term detector damage. The standard deviation over the collected frames is the readout noise. Fig. 3 ▸ shows the pedestal in all three gain modes as well as the readout noise. For all three gain modes the pedestal, as well as the readout noise, is highly correlated within each detector bank. The correlated noise, or *common mode* noise, has previously been documented (van Driel *et al.*, 2020 ▸). van Driel *et al.* showed that this correlated noise can be removed and doing so only offers a small reduction in the readout noise and therefore only provides a substantial improvement for weak signals and single photon counting. Typical TR-XSS experiments flood the detector with photons – this further makes common mode correction challenging as the correction requires unexposed pixels on each bank to characterize the shared common mode with some certainty on a shot-to-shot basis.

**Figure 4:**
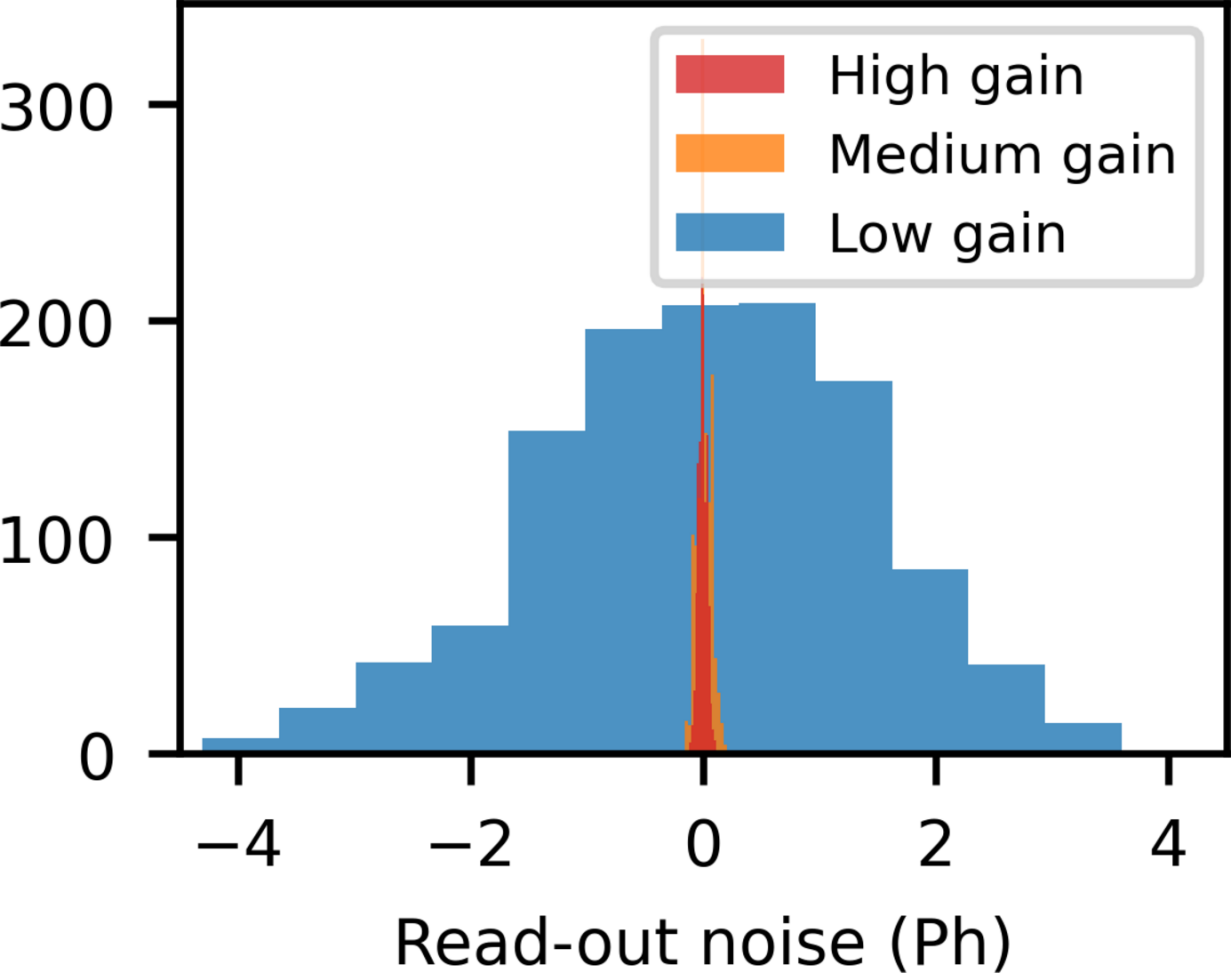
Histograms of the pedestal-subtracted readout under no X-ray exposure in the three different gain modes. The signal has been converted to 9.5 keV photons in order to highlight the signal-to-noise differences between the three gain modes.

At 9.5 keV, the onset of saturation is observed at 6800 (low), 226 (medium) and 68 (high) photons (see Fig. 2 of the supporting information), slightly lower than what has previously been reported (van Driel *et al.*, 2020 ▸). In low gain, the front-end charge-sensitive amplifier uses a larger capacitance, increasing the dynamic range but leading to higher noise. Fig. 4 ▸ shows histograms of pedestal-subtracted readouts from one pixel in different gain modes under no X-ray exposure. The standard deviation of these distributions corresponds to the readout noise, converted to a number of 9.5 keV photons. Table 1 ▸ contains the gain factor, readout noise in both ADU and photons, and the saturation limit for all three gain modes at 9.5 keV.

The large intensity range covered by a liquid scattering signal means that the data quality at the edges of the detector is limited by readout noise in low gain, while the detector saturates in the liquid ring in medium or high gain. In practice, this means that experiments at fixed gain are performed in low gain, sacrificing signal-to-noise to avoid saturation. Alternatively, a choice of different gain modes for different parts of the detector could be deployed, either on a pixel level or on an ASIC level. These ‘mixed’ configurations have been used at several TR-XSS beamlines [Alvra at SwissFEL (Milne *et al.*, 2017 ▸), FXE as Eu-XFEL (Khakhulin *et al.*, 2020 ▸) and XCS/XPP/MFX (Alonso-Mori *et al.*, 2015 ▸; Chollet *et al.*, 2015 ▸; Sierra *et al.*, 2019 ▸) at LCLS]. Whether these gain configurations introduce performance penalties either in the form of non-linearities or changes in intensity-dependent pedestals is not well characterized in the literature. Configuring the gain on an ASIC level is not expected to introduce detector artefacts as they are electronically independent and, as a result, the response of a given ASIC should not be affected by the gain mode of the other. Due to the shape of the liquid ring, the signal-to-noise benefit is, however, larger if it is configured on a pixel level. It has been observed that pixels on the same ASIC of the ePix10k are not entirely independent of their neighbours and exhibit crosstalk. Whether nearby pixels are affected by different gain modes is investigated in the following sections for the ePix10k detector for a typical TR-XSS liquid scattering experiment at the LCLS.

## 3. Methods

### 3.1. Correction

The method employed here was first used to characterize and correct the non-linear gain response of the CSPAD detector (van Driel *et al.*, 2015*b* ▸). This approach utilizes the fact that, except for counting noise, two liquid scattering patterns measured at different X-ray intensities should be linearly proportional to the incoming intensity. By measuring scattering at a range of different X-ray intensities it is possible to characterize the systematic deviation from linearity and create a function that linearizes the gain response. Since the laser-induced signal in TR-XSS is only a small perturbation on top of the bulk liquid signal, the measured pump–probe data can also be corrected with this function, without distorting the pump–probe signal. A more thorough description of the method is available in previous work (van Driel *et al.*, 2015*b* ▸); a cursory overview is presented here.

**Figure 5:**
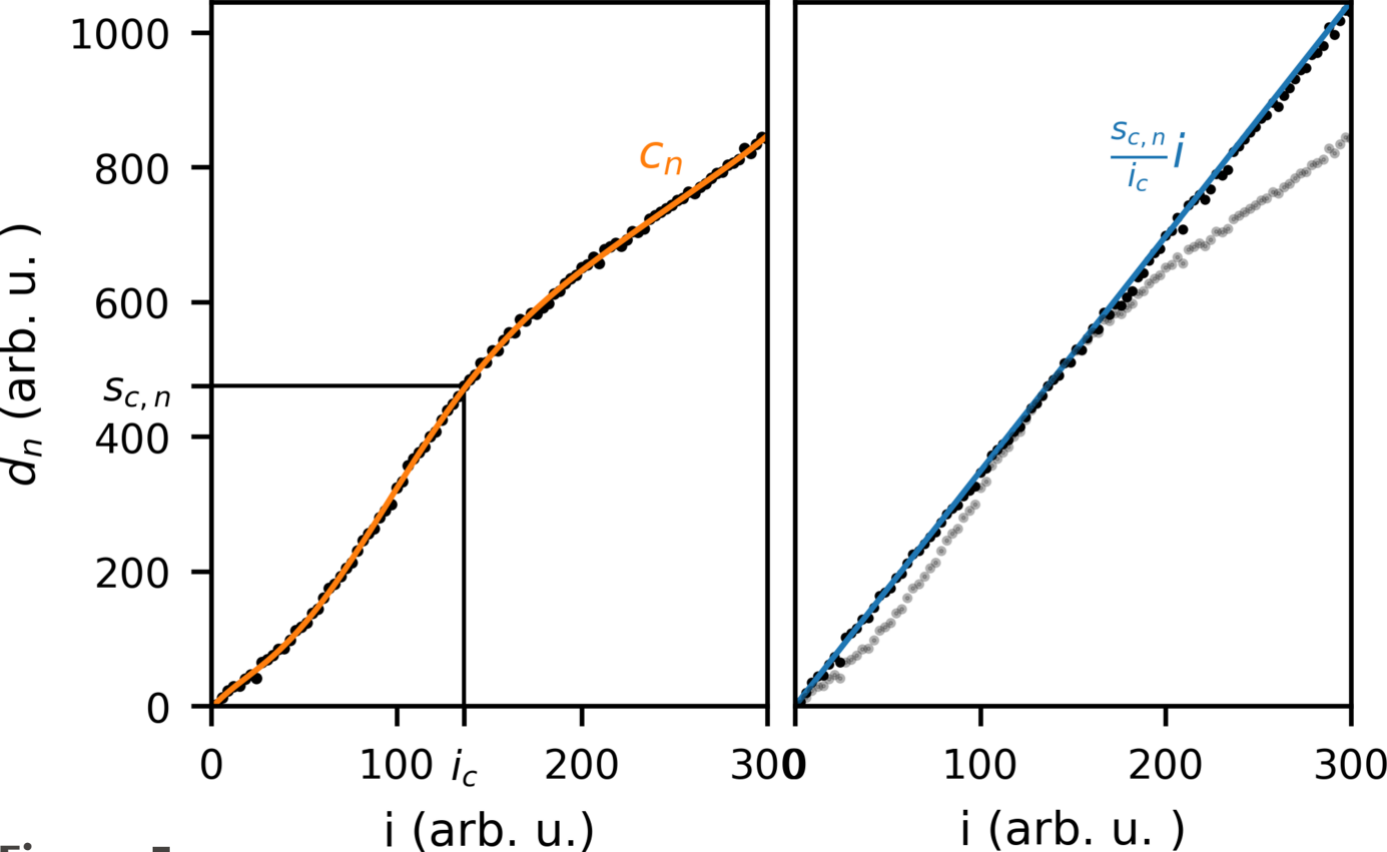
Illustration of the correction procedure. The left figure shows intensity measured by a hypothetical pixel (*d*_*n*_) (black points), along with a polynomial fit *c*_*n*_ (orange). The intensity chosen for re-scaling (*i*_*c*_, *s*_*c*,*n*_) is marked with black lines. The right figure shows the corrected data *s*_*n*_ (black points) compared with (*s*_*c*,*n*_/*i*_*c*_)*i* (blue) and the uncorrected data (grey).

Liquid scattering patterns are measured while varying the X-ray attenuation, and the acquired detector images are binned according to incident X-ray intensity *i*. In previous implementations of the method, *i* was determined as the integrated scattering of a region of the detector. However, to ensure comparability between gain modes, an upstream intensity monitor (IPM5) was used for intensity binning in this work. These approaches are found to be equivalent (see Fig. 1 of the supporting information). The detector response is evaluated as the pixel intensity as a function of incoming X-ray intensity; Fig. 5 ▸ is an illustration of this for the simulated response of a single pixel with index *n*. The measured pixel intensity *d*_*n*_ is approximated by a polynomial fit *c*_*n*_ of *G*th order, and a trusted scattering pattern *s*_*c*_ measured at *i*_*c*_ is determined. *s*_*c*_ is chosen from within the data in an intensity interval where the gain response is approximately linear, but it could in principle also be a simulated signal or a measured reference. The corrected signal *s*_*n*_ as a function of intensity is given by the following expression, 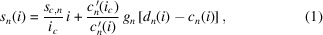
where *c*′(*i*) is the derivative of the polynomial *c*(*i*) with respect to *i*, and *g*_*n*_ is the relative gain between *s*_*c*_ and *d*_*n*_. By imposing the *trusted* slope [first term in equation (1)] and subtracting higher-order polynomial terms (second term), *s*_*n*_ becomes linear, while retaining statistical noise. The order of the polynomial fit required to linearize the data is determined by testing increasing polynomial order, until the difference between *s*_*n*_ and (*s*_*c*, *n*_/*i*_*c*_)*i* is dominated by statistical noise. Fig. 5 ▸ (right) shows a comparison between *s*_*n*_ and (*s*_*c*, *n*_/*i*_*c*_)*i* after correction. Over-fitting is avoided by ensuring that *G* << *M*, where *M* is the number of intensity bins.

The correction function consists of the (*G* + 1)*N*_pixels_ polynomial coefficients as well as the reference scattering pattern *s*_*c*_ and corresponding reference intensity *i*_*c*_. Since *s*(*i*) depends on both *s*_*c*_ and *d*, a unique set of polynomial coefficients must be determined for each sample solvent, detector distance and X-ray photon energy, as well as any other change that influences the intensity distribution on the detector.

The method was developed for correcting experimental scattering data before structural analysis by removing the observed systematic non-linear detector response (van Driel *et al.*, 2015*a* ▸). In the following, we will be using the framework mainly as a tool for characterization, quantifying the magnitude and complexity of the non-linear response by systematically increasing the correction order and visualizing the deviation from the ideal linearity. This allows us to quantitatively compare the *performance* in different gain configurations by comparing the magnitude of the deviation from linear as well as the polynomial order *G* required to linearize the gain response.

### 3.2. Experimental data

The data presented in the following sections represent typical TR-XSS measurements from the liquid standard configuration at LCLS. The data were measured at the XCS endstation, with a 9.5 keV incoming X-ray beam focused to ∼20 µm on a horizontally mounted recirculating 50 µm cylindrical water jet, replenishing the probed volume between the 120 Hz X-ray pulses. The sample chamber is continuously purged with helium to minimize the background scattering from air, and the direct X-ray beam passes through a central hole in the ePix10k large area detector located ∼5 cm downstream from the sample. The detector is read out for each individual X-ray pulse at 120 Hz and covers an angular range around ∼1–60° corresponding to 0–6 Å^−1^ as seen in Fig. 1 ▸(*b*). Upstream of the sample the X-ray intensity is measured using one of the XCS intensity position monitors (IPMs).

## 4. Gain response in fixed gain

The behaviour in fixed low gain was previously characterized by van Driel *et al.* (2020 ▸), who found that the magnitude of the non-linear contributions to the gain were between 0 and 2%, and could be removed with a second- or third-order correction. In this section, this analysis is repeated, to ensure that the results are consistent and to test whether continued use has changed the detector response. Further, the analysis is expanded to include medium gain in order to create a benchmark for the mixed gain modes.

### 4.1. Low gain

**Figure 6:**
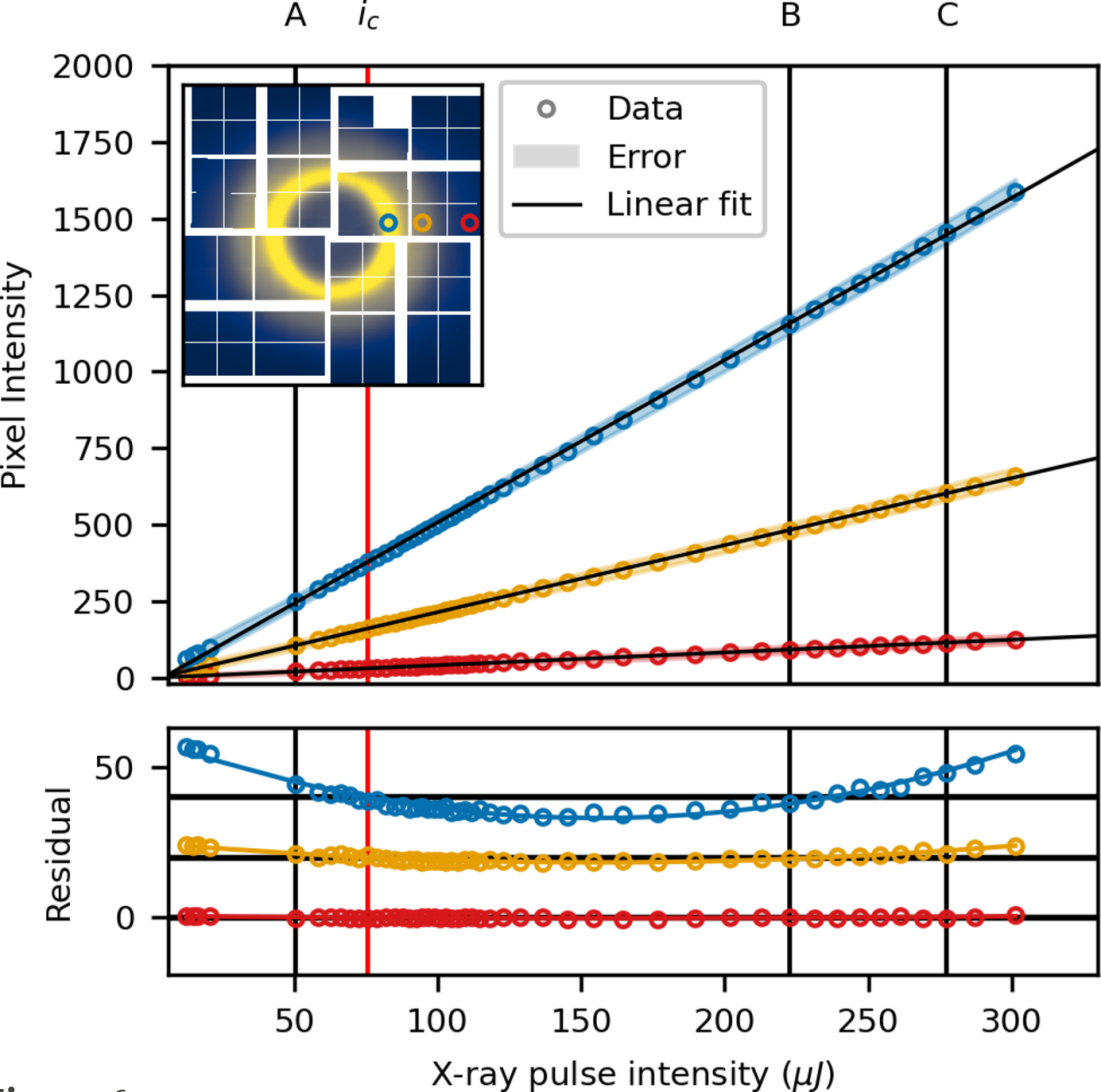
Low gain: per pixel intensity as a function of X-ray intensity for three pixels (shown in the inset). The residual between the measured data and a linear fit is plotted at the bottom. The shaded area denotes the standard deviation in each intensity bin.

Fig. 6 ▸ shows the measured intensity for three individual pixels in low gain, as a function of X-ray pulse intensity, along with linear fits. The chosen pixels are marked in the inset with the colour corresponding to the individual data trace *d*_*n*_. Below, the residual between a linear fit and the data is plotted along with a fitted second-order polynomial. The intensity, *i*_*c*_, around which the data are linearized in the correction procedure, is shown as a red vertical line, along with three reference intensities A, B and C. The residual is largest for the pixel in the area that measures the highest intensity signal, the blue trace taken in the liquid ring. The yellow and red traces are taken at higher scattering angles, corresponding to lower intensity, and the residuals are numerically smaller. The fitted second-order polynomial fits all three traces well.

**Figure 7:**
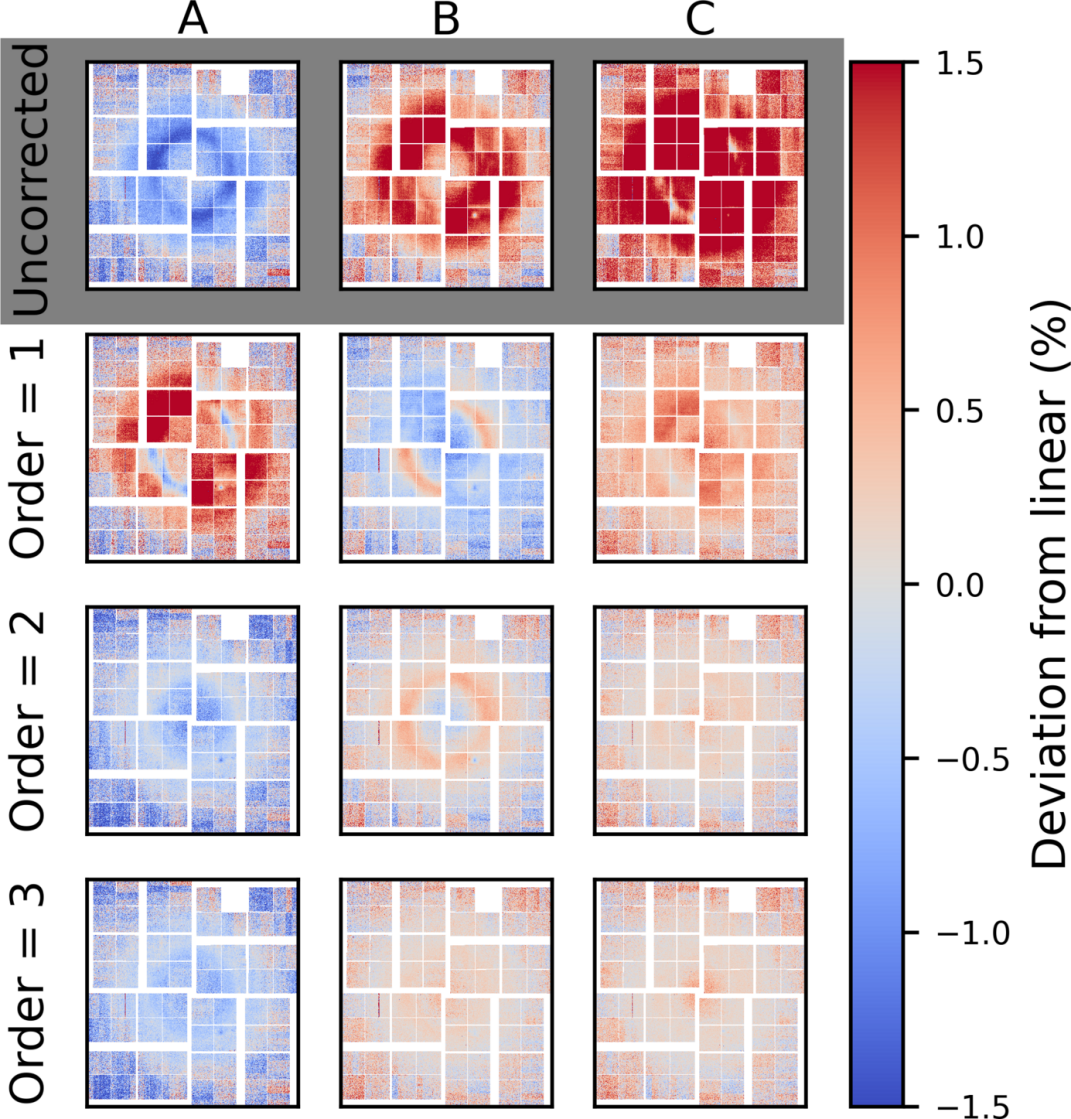
Low gain: the relative deviation from linear, *D*_R_, at three different intensities (A, B and C), plotted for the uncorrected case (grey) and after a first-, second- and third-order correction. The three reference intensities are specified in Fig. 6 ▸.

The magnitude of the non-linear gain components before and after correction is quantified as *D*_R_, the relative deviation from linear. It is calculated as the deviation from the ideal linear case (*s*_*c*_/*i*_*c*_)*i*, relative to *s*_*c*_, 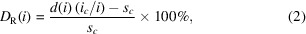
where *d*(*i*), as before, denotes the measured gain response. This value serves as a measure of the magnitude of the non-linear contributions to the gain, *i.e.* how severely are the scattering patterns distorted at a given intensity, with respect to the *trusted* scattering pattern *s*_*c*_. For the purposes of this work, the gain response is deemed linear when *D*_R_ is unstructured and |*D*_R_| ≲ 0.5%. Fig. 7 ▸ shows *D*_R_ at the three reference intensities A, B and C, for increasing correction order *G*.

Before correction (Fig. 7 ▸, top row), the deviation is largest in and around the liquid ring, where the signal is most intense. The magnitude of the deviation increases monotonically between A and B and again between B and C, *i.e.* with intensity-distance from *i*_*c*_, showing that the magnitude of the non-linear gain is monotonic in *i*.

After a first-order correction (Fig. 7 ▸, second row from the top), *D*_R_ decreases from ≳1.5% to ≲0.5% for B and C and becomes visibly asymmetric for A and B, with a different spatial dependence in the upper left and lower right quadrants compared with the other two. The data are deemed linearized after a third-order correction, as *D*_R_ is then dominated by statistical noise. This is similar to what has previously been reported for low gain (van Driel *et al.*, 2020 ▸).

### 4.2. Fixed medium gain

**Figure 8:**
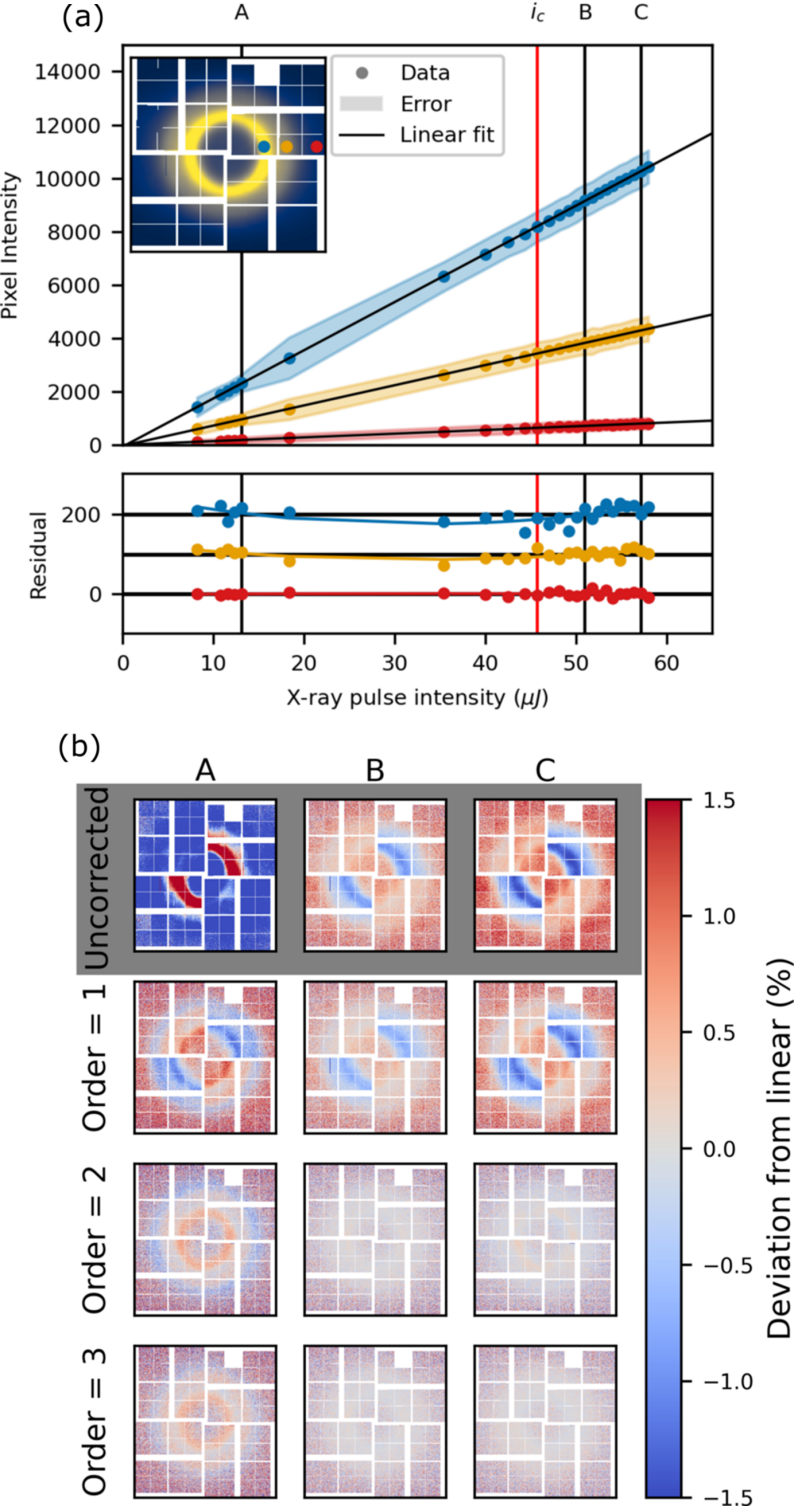
Medium gain: (*a*) per pixel intensity as a function of X-ray intensity for three pixels (shown in the inset). The residual between the measured data and a linear fit is plotted below. The shaded area denotes the standard deviation in each intensity bin. (*b*) The relative deviation from linear at three different intensities (A, B and C), plotted for the uncorrected case (grey) and after a first-, second- and third-order correction. The three reference intensities are specified as vertical black lines in (*a*).

Fig. 8 ▸ shows corresponding plots for medium gain. While scattering was measured beyond the point of saturation, the data shown here have been truncated at 60 µJ in order to avoid saturation, as this is the saturation limit determined (see Fig. 2 of the supporting information). Note that *i*_*c*_ = 45 µJ is closer to B and C, at the high end of the intensity range to avoid the high noise bin at *i* ≃ 18 µJ. The standard deviation in this bin is large due to very sparse sampling in this region. The single pixel behaviour for medium gain in Fig. 8 ▸(*a*) is similar to the behaviour observed in low gain. The gain is less linear at higher intensities (blue trace) and becomes increasingly more linear at lower intensity (orange and red traces). The residual from linear fitting is well described by a second-order polynomial. *D*_R_ [Fig. 8 ▸(*b*)] displays a stronger quadrant dependence than was observed in low gain (Fig. 7 ▸). The magnitude of *D*_R_ is slightly larger than for low gain. Similar to low gain, the non-linear gain components are efficiently removed after a second-order correction as |*D*_R_| ≲ 0.5%.

A similar characterization of the high gain response is presented in see Fig. 3 of the supporting information, but, due to very early saturation onset, *i* = 15 µJ, it is not considered usable for experiments using the liquid standard configuration at LCLS and the full SASE beam. It is therefore excluded from this analysis.

Both medium and low gain show close to linear behaviour throughout the intensity range investigated here. In the following, two different gain configurations, constructed from mixtures of low and medium gain, are investigated.

## 5. Gain response in mixed gain configurations

**Figure 9:**
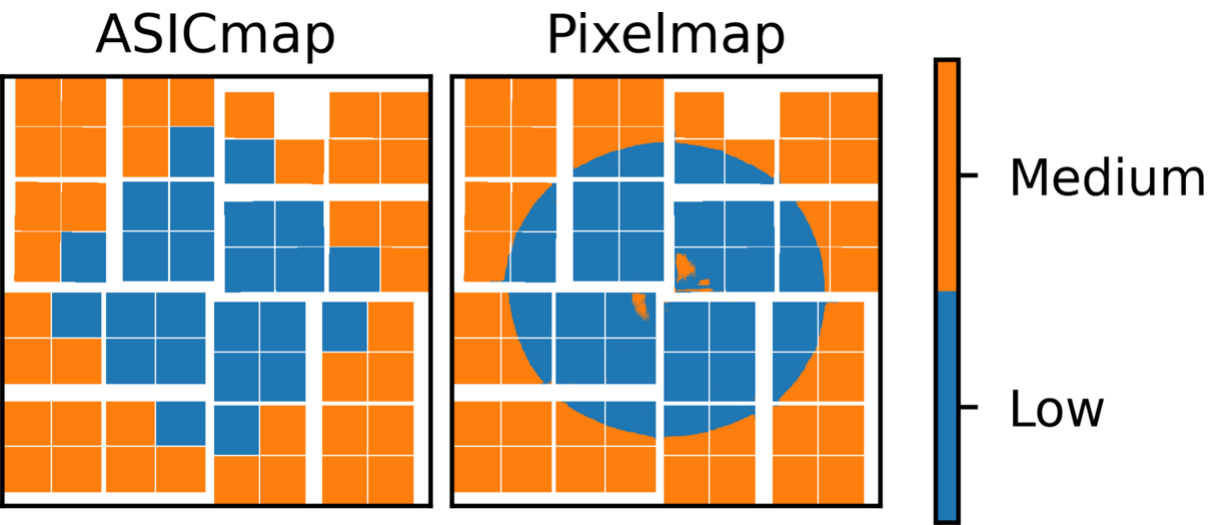
The two mixed gain configurations investigated in Section 5.1. For the left configuration (ASICmap) the gain is set on an ASIC-to-ASIC level. For the right configuration (pixelmap) the gain is set for each pixel individually.

Two different mixed gain configurations have been investigated, in the following referred to as *ASICmap* and *pixelmap*. In the ASICmap configuration the detector gain is configured on an ASIC-to-ASIC level. The ASICmap was constructed to approximate the intensity distribution of the scattering pattern as closely as possible, while keeping a significant pixel fraction in medium gain. As the relative noise contribution is higher in low gain, keeping the number of low gain pixels as low as possible, while avoiding saturation, is the key to improving the signal-to-noise. The left panel of Fig. 9 ▸ shows the ASICmap investigated in this work.

The pixelmap was constructed by measuring an average scattering pattern, at full SASE intensity, in low gain and configuring the detector such that the 40% of the detector that measures the highest intensities is in low gain. The right panel of Fig. 9 ▸ shows the pixelmap configuration studied here.

The medium gain regions for either configurations were not chosen conservatively enough and saturation was observed in both cases. As a consequence of this, the data presented in the following section are truncated at *i* = 150 µJ for the ASICmap and *i* = 250 µJ for the pixelmap, to remove saturation artefacts.

In the following section, all medium gain pixels have been re-scaled by multiplying the measured intensity by the gain factor between low and medium gain (∼1/33, see Fig. 4 of the supporting information). This is done to ease comparison between the gain response of pixels in low and medium gain.

### 5.1. ASICmap

**Figure 10:**
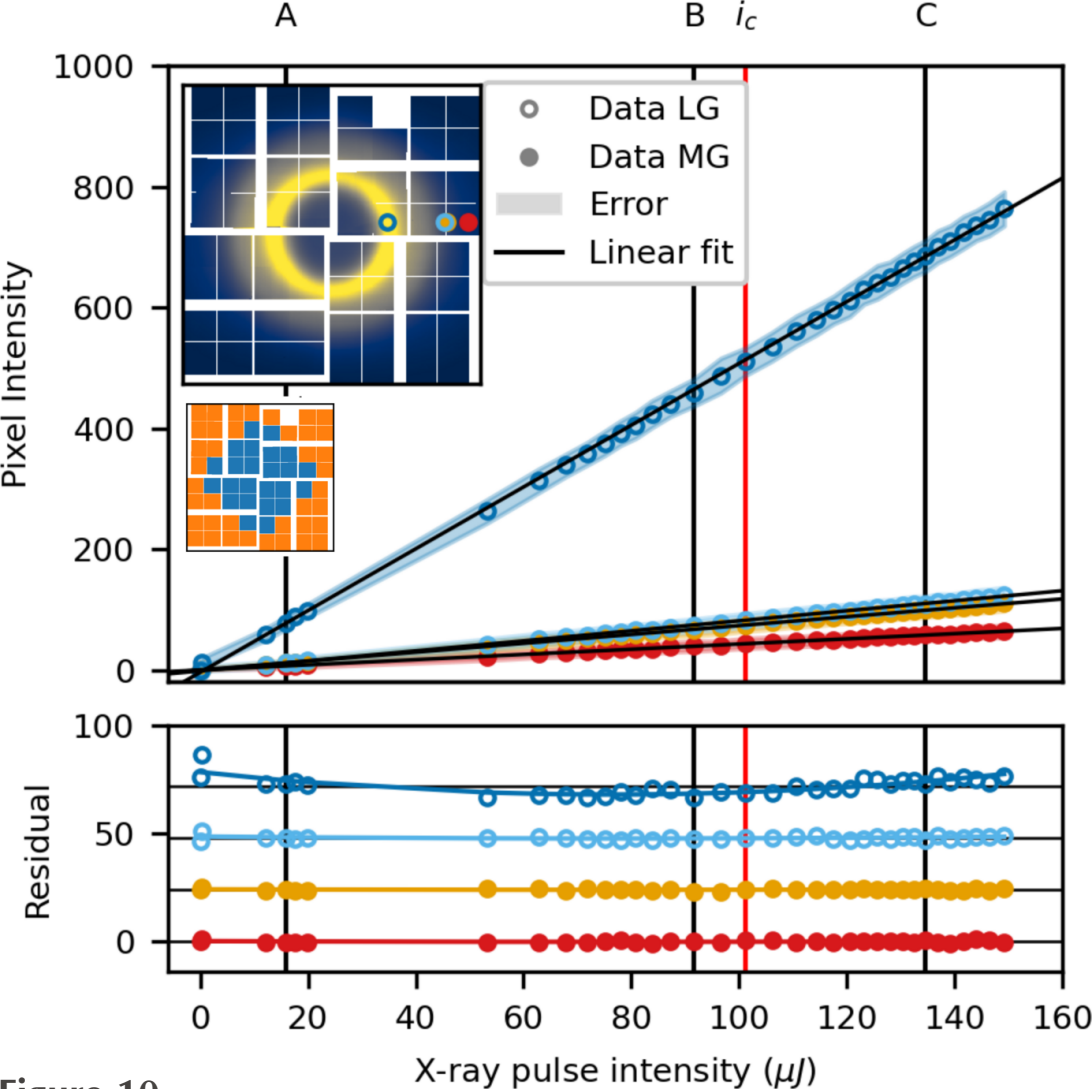
ASICmap mixed gain: per pixel intensity as a function of X-ray intensity, plotted for medium/low ASICmap. Two traces (open circles) are in the low gain region of the detector, the other two (filled circles) are in medium gain. The insets show the total scattering pattern on the detector and the ASICmap used.

The per pixel gain response is plotted as a function of X-ray intensity for the ASICmap configuration in Fig. 10 ▸. The traces are chosen such that the dark blue and red traces are in the low and medium gain regions and far from the gain border, whereas the two intermediate traces are right next to each other on either side of the border between low and medium gain. The residual from linear fitting, for all four pixels, is of the same magnitude as for fixed low and fixed medium gain and only the highest intensity pixel exhibits a stronger second-order non-linearity, consistent with what was observed in low gain (Fig. 6 ▸). The two pixels either side of the gain border (light blue and orange) show no significant increased non-linear gain response, compared with the fixed gain counterparts. This indicates that the gain response in the ASICmap configuration is as linear as what is observed for the fixed gain modes.

**Figure 11:**
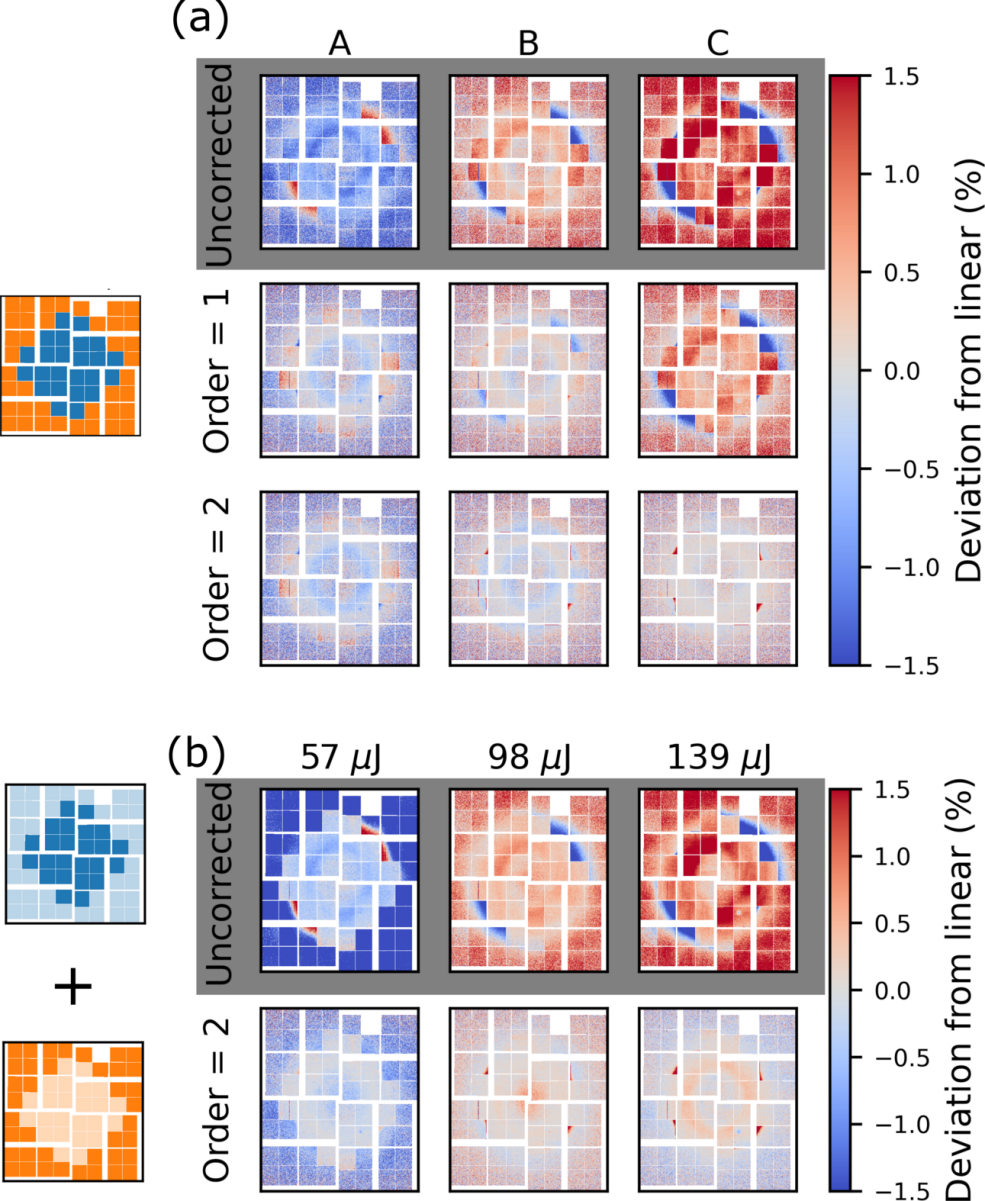
ASICmap mixed gain: the relative deviation from linear (*D*_R_) for medium/low ASICmap at three reference intensities in (*a*). A combination of fixed low and fixed medium gain is plotted (*b*), to highlight differences with the mixed configuration.

Fig. 11 ▸(*a*) shows the relative deviation from linear, *D*_R_, at three reference intensities (A, B and C). To highlight any differences compared with the fixed gain performance, Fig. 11 ▸(*b*) shows the *D*_R_ that would result from the respective pixels performing as they would in fixed gain. This response is assembled from fixed medium/low gain response (Figs. 7 ▸ and 8 ▸) according to the ASICmap. In other words, in the relative deviation shown in Fig. 11 ▸(*b*), the low gain pixels behave as though the entire detector is in low gain and the medium gain pixels behave as though the entire detector is in medium gain. The reference intensities in Fig. 11 ▸(*b*) are chosen such that they are close to A, B and C.

Comparing *D*_R_ in the ASICmap configuration (*a*) with the combination of fixed low and fixed medium gain (*b*), there are no significant differences between the two cases. At B and C, *D*_R_ is unstructured and below 0.5% after a second-order correction. For A, *D*_R_ in the low gain part of (*a*) agrees well with (*b*) both before and after a second-order correction. For the medium gain part of the detector, *D*_R_ is even lower in the ASICmap (*a*) compared with the fixed gain counter part (*b*). This discrepancy is ascribed to different intensity sampling during the two measurements. As the gain response in the ASICmap configuration is consistent with fixed gain, this configuration offers a valid alternative for TR-XSS experiments.

The one significant disadvantage of the ASICmap configuration is the fact that it is not possible to accurately represent the intensity distribution of the ring-shaped liquid scattering pattern with the square ASICs. This means that the beam has to be attenuated to around one-third of the full SASE intensity to avoid saturation on the corners of the innermost medium gain ASICs. Alternatively, the low gain region has to be extended, significantly increasing the number of low gain pixels in the low intensity regions and reducing the signal-to-noise improvement from the mixed gain mode. Alternatively, the gain can be configured on a pixel-to-pixel basis, such as is the case with the pixelmap configuration described in the following section and shown in Fig. 9 ▸.

### 5.2. Pixelmap

**Figure 12:**
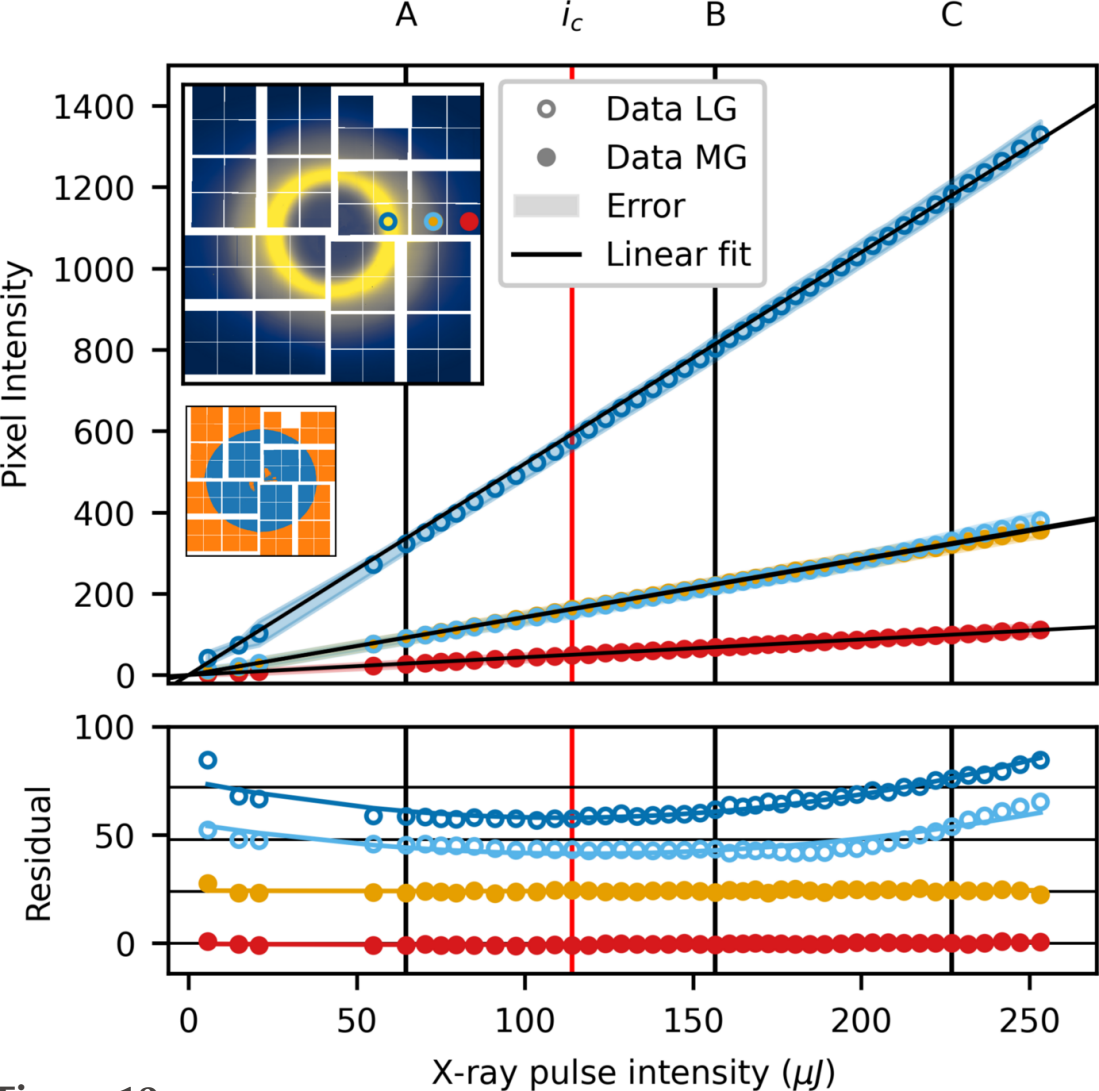
Pixelmap mixed gain: per pixel intensity as a function of X-ray intensity, plotted for medium/low pixelmap. Two traces (open circles) are in the low gain region of the detector, the other two (filled circles) are in medium gain. The insets show the total scattering pattern on the detector and the *pixelmap* used.

Fig. 12 ▸ shows the per pixel intensity as a function of intensity for the medium/low pixelmap gain configuration. The dark blue and red traces are again a low and medium gain pixel far from the gain border. The medium gain trace shows a largely linear behaviour, while the low gain trace, a pixel in the liquid ring, exhibits a second-order contribution to the gain, consistent with what was observed in fixed low gain (Fig. 6 ▸). The two traces at intermediate intensity (orange and light blue) show the response of a medium and a low gain pixel next to each other, on the same ASIC. The medium gain pixel exhibits the same near-linear behaviour as was observed in the fixed medium gain configuration (Fig. 8 ▸). The low gain pixel, on the other hand, shows a much larger residual after linear fitting. In fixed low gain, the response of a comparable pixel (middle trace in Fig. 6 ▸) shows near linear behaviour, with a small second-order component. Examining the residual more closely, it is largely linear up to *i* ≃ 180 µJ, after which it deviates. The response of this pixel is, in other words, largely linear at low intensity but displays super-linear behaviour at high intensity.

**Figure 13:**
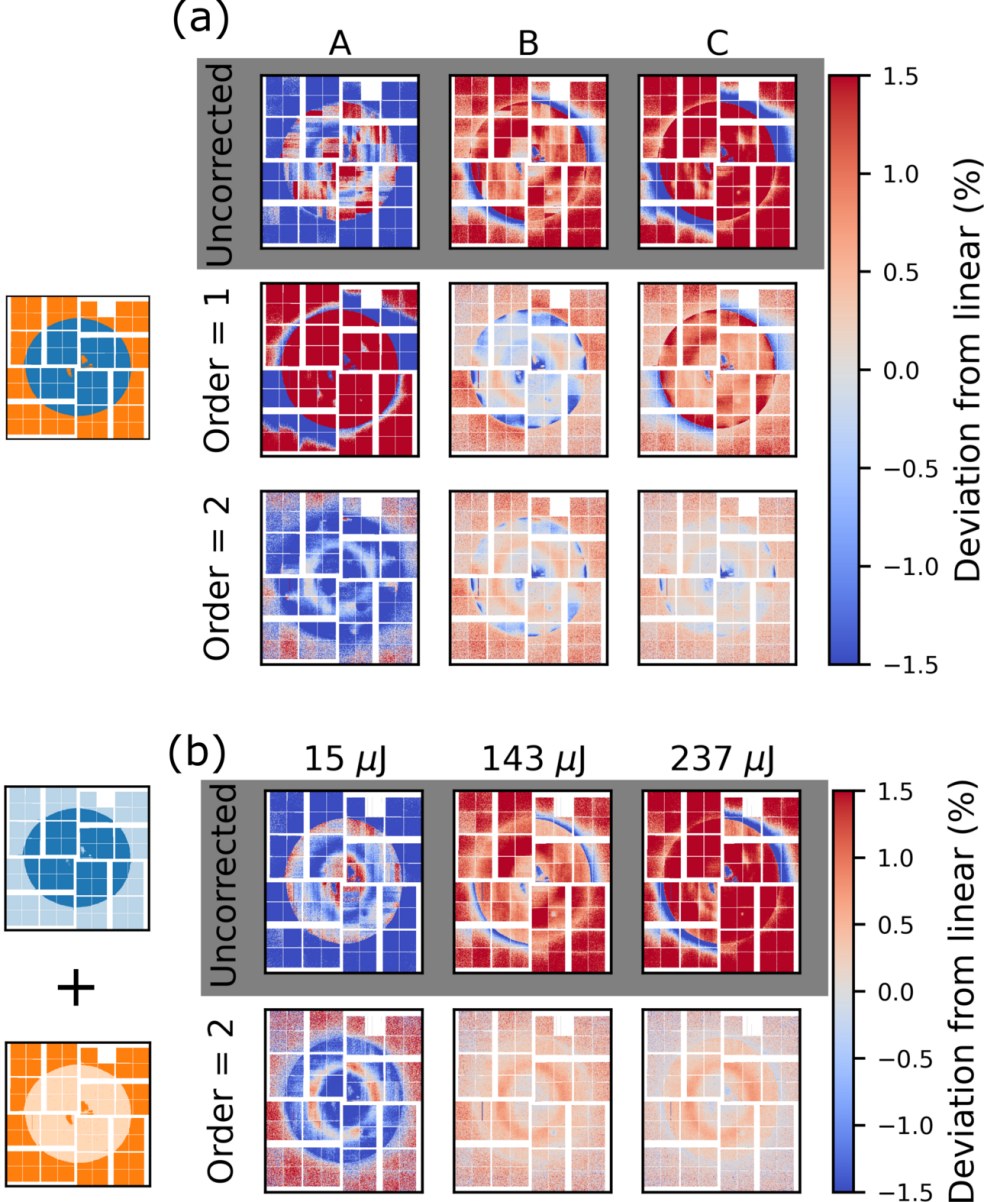
Pixelmap mixed gain: the relative deviation from linear (*D*_R_) at three reference intensities in (*a*). A combination of fixed low and fixed medium gain is plotted (*b*), to highlight differences with the mixed configuration.

**Figure 14:**
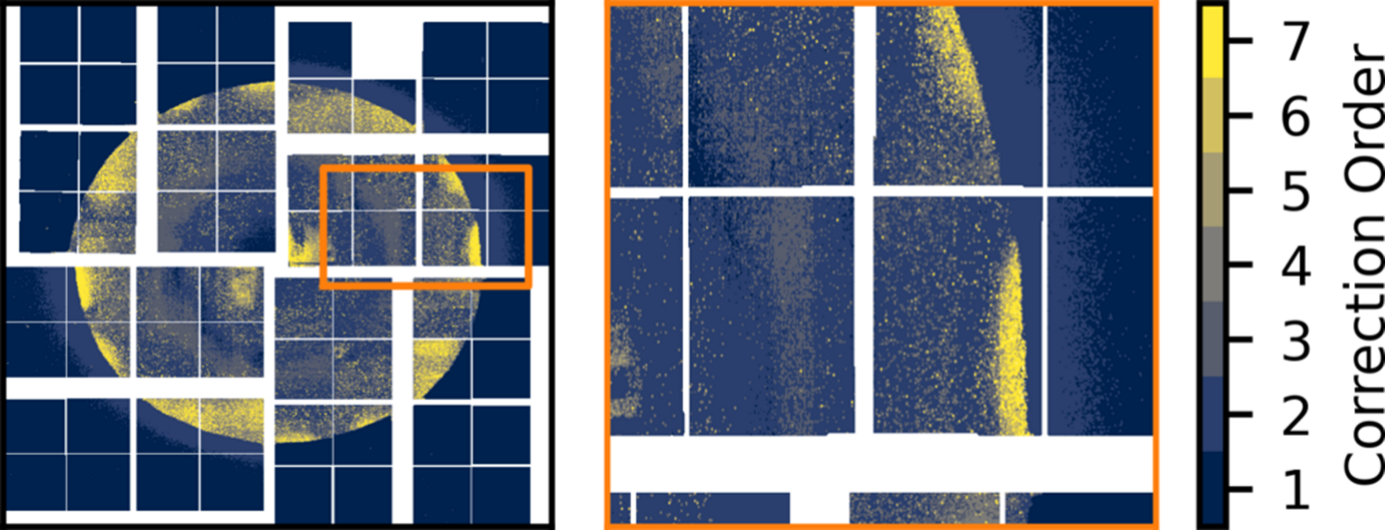
The required correction order to linearize the gain response (left), and a zoom-in on the border between low gain and medium gain (right).

Fig. 13 ▸(*a*) shows the deviation from linear, *D*_R_, at the three intensities A, B and C for uncorrected data as well as for correction orders up to the second order. Fig. 13 ▸(*b*) shows a combination of fixed low and fixed medium gain. While the uncorrected case looks similar between the mixed gain configuration and the fixed cases, after a second-order correction discrepancies arise. Fig. 13 ▸(*a*) shows that, even after a second-order correction, low gain pixels close to the gain border show a significant deviation from linear. Note that this happens only for some of the low gain pixels that share an ASIC with medium gain pixels. To quantify how the behaviour of the pixels depends on the distance to the gain border, the correction order required to linearize each pixel is determined. The data are considered linearized at the lowest correction order for which *D*_R_ is as low or lower than *D*_R_ for the fixed gain mode after a third-order correction. Fig. 14 ▸ shows the required correction order across the entire detector in the medium/low pixelmap configuration (left) as well as a zoom in on pixels on the border between the two gain modes (right).

As outlined in the discussion of Fig. 13 ▸(*a*), the majority of the medium gain pixels are well corrected (|*D*_R_| < 0.5%) with a first-order polynomial. An exception is the innermost pixels in the lower left and upper right quadrant, which require second-order correction (left panel of Fig. 14 ▸). The low gain pixels show more erratic behaviour. Near both the outer border to medium gain as well as near the border at the centre of the detector, the required correction order grows rapidly. The zoom-in shows that, as the switching border is approached from the low gain side, the required correction order increases from second order up to sixth order very close to the low/medium gain boundary, while the required correction order of the medium gain pixels is entirely unaffected.

Mixing gain modes on the same ASIC negatively affects the linearity of the low gain pixels; the incurred penalty is, however, rather small (∼1–2% increase in non-linear gain response). It appears that the decrease in linearity of the gain response near the gain boundary sets in at higher intensities (∼180 µJ). Since the dynamic range in low gain allows for several times this intensity, the departure from linearity is likely caused by the high intensity on nearby medium gain pixels. It is therefore plausible that this effect can be removed by increasing the extent of the low gain region. For this purpose, a configuration procedure has been developed. It is designed to minimize the magnitude of the non-linear contributions to the low gain along the gain boundary, while maximizing the signal-to-noise. The following section outlines this procedure.

#### 5.2.1. Pixelmap configuration

**Figure 15:**
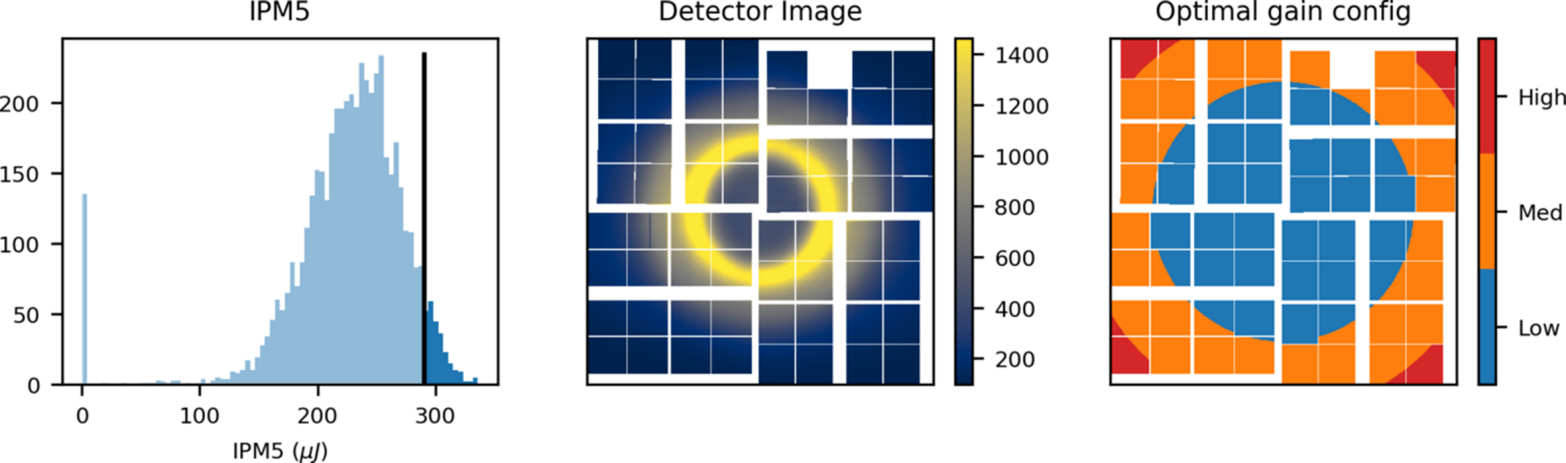
Output from the pixelmap configuration script. For a low gain scan, the 0.95 intensity quantile is found based on the IPM5 monitor (left). Based on the corresponding detector images an average detector image is calculated (middle) and a corresponding gain configuration is suggested (right), based on the set saturation threshold in ADU. For this particular configuration, the threshold was set to 11000 ADU, after pedestal subtraction.

Whether a pixel should be in low or medium gain for a given measurement should be decided based on the expected intensity in that pixel. In principle the intensity can be estimated by measuring a single scattering pattern. However, due to SASE fluctuations, a single detector image is not very likely to represent the typical intensity distribution on the detector. On the other hand, generating the configuration from a statistical average of measured scattering patterns will result in a configuration that is prone to saturation during high intensity exposures. For the proposed configuration scheme, a measurement should be performed in low gain. A detector image, representing high intensity scattering events (Fig. 15 ▸, middle), is then produced from an average of the shots in the 95th intensity percentile (Fig. 15 ▸, left). The optimal gain setting for each pixel is chosen as the highest one that avoids saturation. A conservative estimate for saturation is 11000 ADU after pedestal subtraction (see Fig. 2 of the supporting information). The detector is split into radial bins and the gain in each of these bins is set as the lowest gain estimate within each of them. This minimizes the perimeter of the gain border, which is where non-linear gain arises in the pixelmap (Fig. 12 ▸). The suggested gain for each pixel is shown in Fig. 15 ▸ (right); note that only two different gains can be chosen for the actual detector configuration.

This pixelmap configuration leaves ∼65% of the pixels in medium gain, compared with ∼50% for a corresponding ASICmap configuration that avoids saturation. The optimal choice of gain mode for a given TR-XSS experiment will depend on the sample solvent, the X-ray energy, as well as whether the beam is attenuated or monochromated and can be determined with the method presented here.

## 6. Conclusions

The analysis presented here shows that the gain response in all three fixed gain modes of the ePix10k detector is largely linear, with small second- and third-order non-linear gain components, in good agreement with what was previously found for low gain (van Driel *et al.*, 2020 ▸). The authors note that the timing of the upper right and lower left quadrant was slightly mistimed, resulting in an overall worse detector response, that has since been addressed. This timing issue does not affect the conclusions regarding crosstalk and optimal choice of mixed gain configurations but does amplify the magnitude of the non-linear gain response in these quadrants. The detector response is characterized in two mixed gain modes – one where the gain mode is set on an ASIC-to-ASIC level and one where it is set on a pixel-to-pixel level ASICmap and pixelmap. The ASICmap configuration shows no increase in the polynomial order or magnitude of the non-linear gain components compared with the fixed gain modes. However, due to the mismatch between the square ASICs and the circular liquid scattering pattern, creating an ASICmap configuration that avoids saturation limits the signal-to-noise benefit from the mixed gain mode. The gain response in the pixelmap configuration is similar to the response in the fixed gain modes, with the notable exceptions of a subset of the low gain pixels that are on the same ASIC as medium gain pixels. These pixels show a significant increase in the polynomial order needed to correct the gain response at high X-ray intensity. The non-linear contributions to the gain response can be removed using the method detailed here and by van Driel *et al.* (2015*b* ▸), but to avoid it in the future a method for optimal pixelmap configuration is presented in Section 5.2.1.

## Supplementary Material

PDF document containing additional considerations. DOI: 10.1107/S1600577524012219/yi5164sup1.pdf

## Figures and Tables

**Table 1:** Key values for the three gain modes of the ePix10k detector

Gain	Relative gain factor	Noise (ADU)	Noise (photons)	Saturation (photons)
Low	1	2.4	1.48	6800
Medium	33	3.2	0.07	226
High	100	6.4	0.04	68
