# Supplementary material for: Mixed gain detector configurations for time-resolved X-ray solution scattering
Source: J Synchrotron Radiat. 2025 Feb 6;32(Pt 2):355–64. doi: 10.1107/S1600577524012219 (PMC11892888; doi:10.1107/S1600577524012219)
Supplement: Supplementary file 1 [file s-32-00355-sup1.pdf]

JOURNAL OF SYNCHROTRON RADIATION

Volume 32 (2025)

Supporting information for article:

Mixed gain detector configurations for time-resolved X-ray solution scattering

Morten Lunn Haubro, Joseph Pon, Philip Adam Hart, Kristoffer Haldrup and Tim Brandt van Driel

Supporting Information for "Mixed Gain Detector Configurations for Time Resolved X-ray Solution Scattering"

1. Additional Considerations

Several considerations play a role in configuring and evaluating the detector performance. While the general behavior can be observed without looking into these additional aspects, it is important to keep some choices in mind as they may affect the outcome or bias the results. While such biases may be small, they may have larger implications when extracting small signals or when filtering or sorting the data in which case fluctuations can turn into systematic errors.

1.1. Choosing an Intensity probe

To quantify the gain response the measured scattering patterns are binned according to an X-ray intensity probe i, which should be proportional to the intensity in each pixel. In previous implementations of this method, an integrated region of interest (ROI) near the edge of the detector was used. The problem with the choice of such an i probe is that it depends on the gain mode, and hinders direct comparison between gain modes. Here, we use the X-ray pulse intensity as measured by the intensity position monitor IPM5 to readily compare the detector performance across gain modes. Figure 1 shows the correlation between IPM5 and the median intensity in the chosen ROI (shown in the insert). This correlation is observed to be linear.

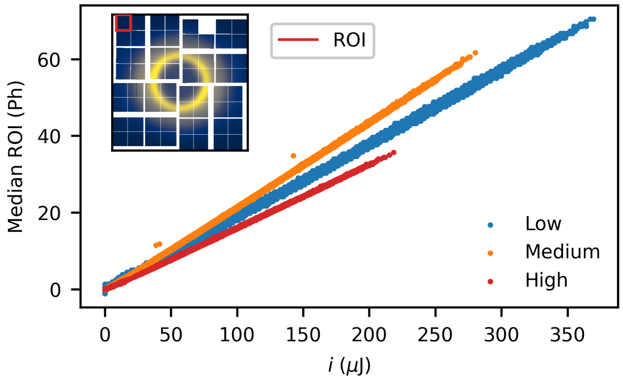

Fig. 1. Median pixel intensity in a region of interest (ROI) plotted as a function of the Intensity Position Monitor (IPM5), here denoted i. The correlation is plotted for all three gain modes, high (red), medium (orange), and low (blue). The median ROI in high and medium gain has been divided with the nominal gain factor of 100 and 33 for comparability with low gain.

1.2. Saturation Considerations

The ePix10k detector has three fixed gain modes high, medium, and low. The relative gain factors between them is (100, 33, and 1). As outlined in the introduction, increasing the gain reduces the dynamic range of the detector, increasing the risk of saturation. It is therefore important to consider the effective usable dynamic range of the detector in each gain mode. As the pixels are read out as 14 bit integers the maximum value a pixel can have is 16383 ADU. Including the pedestal, this value corresponds to roughly 8200 photons in low gain, 270 in medium gain, and 80 in high gain at 9.5 keV (van Driel *et al.*, 2020). Figure 2 shows the mean intensity in the liquid ring, after pedestal subtraction, as a function of intensity in medium and high gain. The mean intensity in the liquid ring grows linearly with X-ray intensity, until around $\sim$ 11000 ADU, where it begins to taper off towards saturation. To avoid saturation and non-linear effects arising in the vicinity of saturation, detection should ideally be kept below this limit, bringing the effective detection limit down to around 6800, 226, and 68 photons/pixel/pulse in each of the three gain modes respectively. This

corresponds to an incident X-ray intensity of 60 μJ and 20 μJ for medium and high gain respectively, for the experimental data presented here. High and medium gain data presented in this work has been truncated at these values to avoid saturation.

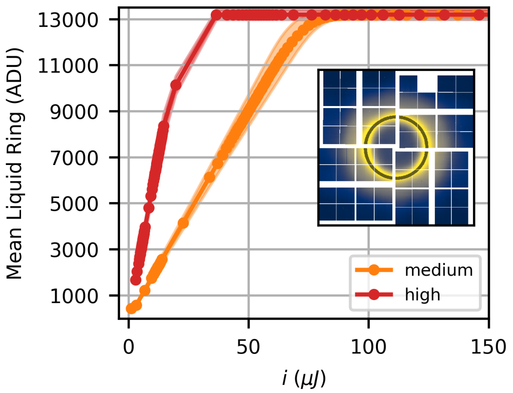

Fig. 2. Mean intensity in the mask shown in the insert for both medium (orange) and high (red) gain. The shaded area corresponds to the standard deviation.

2. Detector Response in High Gain

The per pixel gain response in high gain is plotted in figure 3 (a). The response is well fitted by a first order polynomial, even in the highest intensity regions. This is confirmed when looking at the deviation from linear (b), note that the range on the colorbar has been increased by a factor of 2 compared to medium and low gain. As the non-linear contributions to the gain are removed effectively by a 1st order correction, it appears that the gain response in high gain is largely linear throughout the intensity range investigated here. The measured effect can be interpreted as the presence of an additional *effective* pedestal. As it is gone after a first order correction there is no evidence that it is intensity dependent. Examining the shape of D_R in the uncorrected case in high gain (figure 3 (b)), a distinct spatial pattern emerges, the pattern appears highly correlated with the bank structure of the detector (see figure 2 in the main text). It should be noted that high gain was measured for around 1/4 of

the time when compared to low and medium gain, which contributes to the relatively higher statistical noise.

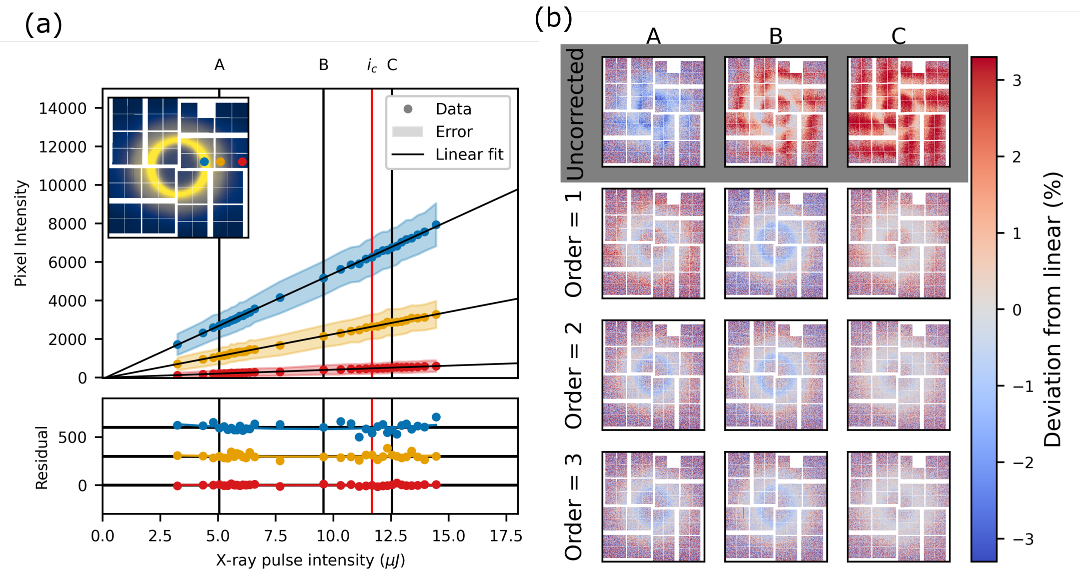

Fig. 3. High gain: a) Per pixel intensity as a function of X-ray intensity for three pixels (shown in the insert). The residual between the measured data and a linear fit is plotted below. The shaded area denotes the standard deviation in each intensity bin. b) D_R at three different intensities (A, B, and, C), plotted for the uncorrected case (grey) and after a first, second and third order correction. The three reference intensities are specified as vertical black lines in a).

3. Relative Gain

To compare and analyze the signals measured in mixed gain configurations, pixels in different gain modes need to be rescaled according to the relative gain between them. While the absolute gain has been determined for each of the three gain modes (van Driel *et al.*, 2020), these values may vary across the detector or even change as a result of time, usage or radiation damage. The relative gain between two gain modes can, be calculated as the ratio between the slopes in the two gain modes after correction. Figure 4 shows the relative gain between the three gain modes after a 3rd

order correction.

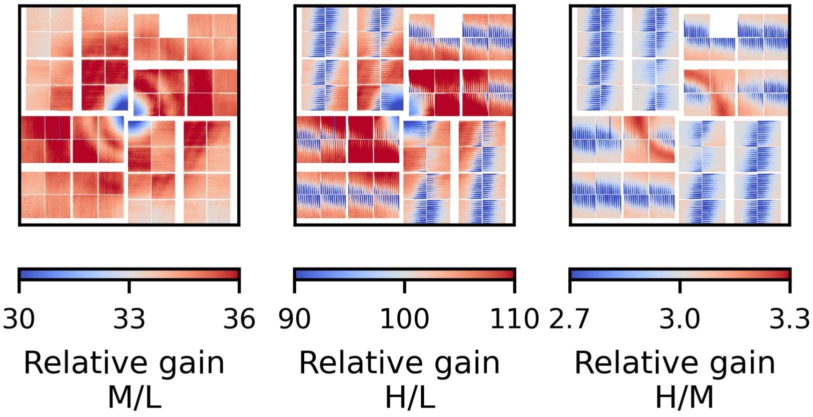

Fig. 4. The relative gain plotted across the detector. The nominal relative gains are 33, 100 and 3. Each plot shows a deviation of roughly 10 % across the detector.

As outlined in the introduction, the nominal relative gain is 33 between medium and low gain, 100 between high gain and low gain, and thus 3 between high gain and medium gain. For all three combinations, we observe a deviation from design specifications of up to 10 % across the detector. For the medium/low combination (right), the deviation from the nominal gain is largest at intermediate scattering angle, i.e. in and around the liquid ring. The deviation at low scattering angle, right around the beamcenter, can be ascribed to changes in air scattering between the low and medium gain measurement. Air scattering, shows up at low scattering angles and as the signal from the liquid sample is relatively small in this area, even a small variation in air scattering may cause a relatively large change in the scattering. Both the High/Low and High/Medium relative gain is dominated by the same spatially distinct pattern that was observed in the uncorrected deviation from linear in high gain, (figure 3 (b), top row).

The very early onset of saturation in high gain makes high gain unsuited for liquid scattering experiments with the full SASE beam at the LCLS. For this reason, as well

as because of the systematic deviations from nominal gain, we chose to exclude high gain from the rest of the investigation and focus on combinations of medium and low gain.

References

van Driel, T. B., Nelson, S., Armenta, R., Blaj, G., Boo, S., Boutet, S., Doering, D., Dragone, A., Hart, P., Haller, G., Kenney, C., Kwaitowski, M., Manger, L., McKelvey, M., Nakahara, K., Oriunno, M., Sato, T. & Weaver, M. (2020). *Journal of Synchrotron Radiation*, **27**, 608–615.
